# Novel silicon-based material decomposition images in diagnosis of pancreatic ductal adenocarcinoma: comparison with iodine-based and 50-keV virtual monoenergetic images

**DOI:** 10.1007/s11604-025-01856-9

**Published:** 2025-08-22

**Authors:** Yoshifumi Noda, Mayu Hattori, Nobuyuki Kawai, Tetsuro Kaga, Akio Ito, Takuma Ishihara, Toshiharu Miyoshi, Yukiko Takai, Masashi Asano, Hiroki Kato, Fuminori Hyodo, Avinash R. Kambadakone, Masayuki Matsuo

**Affiliations:** 1 Department of Radiology, Gifu University, 1-1 Yanagido, Gifu 501-1194, Japan; 2 Department of Radiology, Massachusetts General Hospital Harvard Medical School, 55 Fruit Street, White 270, Boston, MA 02114 USA; 3 Innovative and Clinical Research Promotion Center, Gifu University Hospital, 1-1 Yanagido, Gifu, 501-1194 Japan; 4 Department of Radiology Services, Gifu University Hospital, Gifu 501-1194, Japan; 5 Department of Pharmacology, Graduate School of Medicine, Gifu University, 1-1 Yanagido, Gifu 501-1194, Japan; 6 Center for One Medicine Innovative Translational Research (COMIT), Institute for Advanced Study, Gifu University, 1-1 Yanagido, Gifu 501-1194, Japan

**Keywords:** Multidetector computed tomography, Pancreatic cancer, Image reconstruction

## Abstract

**Objectives:**

To identify the optimal material decomposition (MD) images for diagnosis of pancreatic ductal adenocarcinoma (PDAC) and evaluate the added value of the MD image to 50-keV virtual monoenergetic images (VMIs) by comparing with iodine-based images and 50-keV VMIs.

**Methods:**

This retrospective study included patients who underwent pancreatic protocol dual-energy CT (DECT) between February 2019 and May 2023. First, a radiologist evaluated 702 image datasets generated using 27 different materials to identify the top three MD images which provided maximum contrast difference between normal pancreas and PDAC, and subsequently, the best MD image was selected based on z value and image quality by four radiologists. Then, another four radiologists independently interpreted the conventional image dataset (iodine-based images and 50-keV VMIs) and new optimal image dataset (optimal MD images and 50-keV VMIs), and graded the presence or absence of PDAC. The sensitivity, specificity, positive predictive value (PPV), negative predictive value (NPV), and accuracy were compared between the two image datasets using generalized estimating equations.

**Results:**

Overall, 110 patients (median age, 73 years; 63 men) were included. Among them, 67 patients (61%) had pathologically proven PDAC, and the optimal MD image selected was Silicon/Struvite. The optimal image dataset had higher specificity (88% vs. 81%; *P* = 0.006), PPV (93% vs. 89%; *P* < 0.001), and accuracy (94% vs. 92%; *P* = 0.01) than the conventional image dataset. No difference was found in the sensitivity (*P* = 0.34) and NPV (*P* = 0.33) between the two image datasets.

**Conclusion:**

Silicon/Struvite images provided high contrast difference between normal pancreas and PDAC and higher diagnostic performance for diagnosis of PDAC in combination of 50-keV VMIs compared to iodine-based images and 50-keV VMIs.

## Introduction

Pancreatic protocol CT, including pancreatic and portal venous phases, is the most widely used and preferred imaging modality for the diagnosis and staging of pancreatic ductal adenocarcinoma (PDAC) [1]. The pancreatic phase provides the most optimal contrast difference between hypo-enhancing PDAC and surrounding enhancing normal pancreatic parenchyma. However, small (< 2 cm), iso-attenuating, and non-contour deforming tumors can be missed on pancreatic protocol CT [2, 3]. Until now, various techniques have been developed to enhance diagnosis of PDAC using pancreatic protocol CT [4].

The improved material characterization capabilities of dual-energy CT (DECT) have been widely investigated to enhance detection and characterization of pancreatic lesions [5]. DECT acquisition enables creation of multiple different types of images which enhance tissue characterization, such as virtual monoenergetic images (VMIs), material decomposition (MD) images, and virtual non-contrast images [6]. VMIs at low energy levels (40–55 keV) have been shown in several studies to enhance detection of PDAC by improving conspicuity, diagnostic performance, and radiologists’ confidence in detecting PDAC [4, 7–11].

Among the MD images, the most widely used image datasets are the iodine-based images which allow quantitative and qualitative assessment of tissue enhancement. A combination of low energy VMIs (50 keV) and iodine-based images is routinely acquired as part of DECT protocol for the pancreas [5]. For the iodine-based images, several researchers have investigated iodine concentration as a quantitative imaging biomarker for the assessment of therapeutic response [12], disease characterization [13, 14], and determination of lymph-node metastasis [15, 16]. While DECT acquisition enables generation of a wide range of MD images, there are limited data on other MD images specifically in the diagnosis and characterization of pancreatic lesions. Therefore, the purpose of our study was to identify the optimal MD images for diagnosing PDAC and then evaluate the added value of the optimal MD images when used in combination with 50-keV VMIs for diagnosing PDAC.

## Materials and methods

### Patients

This was a retrospective study approved by our institutional review board, and written informed consent was waived. Data generated by the authors or analyzed during the study are available at Gifu University Hospital. Between February 2019 and May 2023, 122 consecutive patients with known or suspected pancreatic disease based on previous CT examinations, clinical symptoms, and/or blood test values who underwent pancreatic protocol DECT were identified. The inclusion criteria are adult patients (≥ 18 years) and scanned by Revolution CT with Apex edition (GE HealthCare). The exclusion criteria were patients who have severe pancreatic atrophy, receive reduced contrast material dose, or have malignant lesions other than PDAC, and lack of raw DECT data.

### Dual-energy CT technique and contrast injection

All examinations were performed with a rapid kilovoltage-switching DECT scanner (Revolution CT with Apex edition). The pancreatic protocol DECT imaging parameters were tube voltage (80/140 kVp), noise index (7.0 Hounsfield unit; HU) at 5-mm slice collimation based on adaptive statistical iterative reconstruction-Veo of 40%, tube current (variable; GSI Assist; GE HealthCare), detector configuration (128 detectors with 0.625-mm-section thickness), beam collimation (80 mm), rotation time (0.5 s), pitch (0.581:1), scan field of view (large body), and display field of view (38 cm). The raw DECT data at 1.25-mm-section thickness with 50% overlap were reconstructed adaptive statistical iterative reconstruction-Veo of 40%. Reconstructed images at 1.2-mm thickness in the coronal plane were also available.

Intravenous iodinated contrast material containing a 300-mg iodine per milliliter (iopamidol, Iopamiron 300®, Bayer HealthCare or iohexol, Omnipaque 300®, GE Healthcare Pharma) adjusted for the patients’ body weight (600 mg of iodine per kg) was injected over a fixed duration of 30 s. Real-time fluoroscopic monitoring scan (140-kVp, 10 mA) was initiated 10 s after administering contrast injection. Diagnostic CT scans were performed with additional delays of 20 and 60 s for the pancreatic and portal venous phases, respectively, after a bolus-tracking program (SmartPrep; GE HealthCare) detected an 80-HU threshold in the abdominal aorta.

### Identification of the optimal material decomposition images

The DECT images were transferred to Advanced Workstation server 4.7 (GE HealthCare). Twenty-seven materials were used for reconstructing MD image datasets after installing mass attenuation coefficient csv file of all materials which were provided by the vendor (Table 1). One MD image dataset consists of two materials, each of which has an opposite combination. Therefore, a total of 702 (27 × 26) image datasets were obtained for the final patient cohort.

**Table 1:** Materials used for reconstructing material decomposition image datasets

Material
Octacalcium phosphate (OCP)
Brushite
Silicon
Calcium
Phosphorus
Struvite
Water
Monosodium urate
Calc measured
Muscle
Cystine
Iodine
Cortical bone
Bilirubin
Calcium pyrophosphate dehydrate deposition (CPPD)
Fat
Hydroxyapatite (HAP)
Uric acid
Tricresyl phosphate (TCP)
Bone
Calcium oxalate mono-hydrate (CaOxMono)
Calcium chloride (CaCl_2_)
Blood
Calcium phosphate
Natrium
Sodium chloride (NaCl)
Calc bone

A study coordinator (Y.N., with 12 years of experience in abdominal radiology) placed circular regions of interest (ROIs) on the normal pancreas and PDAC on the pancreatic phase 50-keV VMIs in the patients with PDAC. For the normal pancreas, the ROI was placed at a spared portion of pancreatic parenchyma in patients with PDAC at the head/uncinated process or a downstream segment from PDAC in patients with PDAC at the body/tail. For PDAC, the ROI was placed as much of the lesion as possible on images showing the maximum PDAC diameter while avoiding artifacts and large vessels. The ROI placed on the 50-keV VMIs were automatically transferred to a similar location on the other VMIs datasets at 40-, 45-, 55-, 60-, 65-, and 70-keV levels and all 702 MD image datasets.

Subsequently, the contrast difference between normal pancreas and PDAC was calculated using the following equation: contrast difference = [(CT attenuation or concentration of normal pancreas − those of PDAC)/CT attenuation or concentration of normal pancreas] × 100 (%). The top three image datasets showing maximum contrast difference were selected, and then, four radiologists selected the best one based on the *z* value and image quality for diagnostic acceptability in consensus. The *z* value is a statistical measurement that describes the relationship between the mean value and standard deviation (SD) and normalizes the measurements with consideration for not only the mean value but also variability. The greater absolute figure of the *z* value represents higher mean value and less variability with stability compared to the small absolute figure of the *z* value.

### Image analysis

The pancreatic phase iodine-based (Iodine/Water) images and 50-keV VMIs (conventional image dataset) and the pancreatic phase optimal MD images and 50-keV VMIs (optimal image dataset) were independently reviewed in a random order by four radiologists who are different from four radiologists mentioned in the previous section, which included two expert radiologists (T.K. and N.K., with 6 and 11 years of experience in abdominal radiology, respectively [expert]) and two trainees (M.H. and A.I., with 1 and 3 years of experience in radiology [trainee]) blinded to clinical information, material pair’s name, and final diagnosis. The preset window settings for the 50-keV VMIs were fixed at 490-HU width and 90-HU level [17], those for the iodine-based images were fixed at 150-HU width and 50-HU level (GE HealthCare’s default setting), and for the optimal MD images identified were fixed at 1,500-HU width and – 550-HU level (lung window). The radiologists were allowed to adjust the window settings at their own discretion during the interpretation.

The radiologists independently graded the presence or absence of PDAC using a five-point scale [4]: 5, definitely present (unequivocal visualization of PDAC distinct from surrounding nontumorous pancreas); 4, probably present (likely presence of an indistinct PDAC along with secondary features such as duct cutoff); 3, equivocal (indistinct PDAC may be present and secondary features such as duct cutoff are lacking); 2, probably absent (poor visualization of pancreatic mass from surrounding nontumorous pancreas); and 1, definitely absent (no pancreatic tumor identified). A confidence rating of 3–5 was considered indicative of the presence of PDAC.

### Reference standard for PDAC

All PDACs were confirmed by ultrasound-guided fine-needle aspiration and pathological diagnoses. Among them, 30 PDACs were surgically resected and pathology obtained. Absence of PDAC was confirmed by the study coordinator’s interpretation; no progression on follow-up imaging studies, including CT and MRI for at least 6 months; or negative results on ultrasound-guided fine-needle aspiration.

### Statistical analysis

To ensure adequate statistical power for detecting differences in specificity between the conventional and optimal image datasets, the required sample size was calculated using Monte Carlo simulations, factoring in a fixed number of four reviewers. First, we assumed patient-level correlations ranging from 0.01 to 0.5 and generated random numbers from a bivariate Bernoulli distribution, assuming a specificity of 80% for the conventional image dataset and 90% for the optimal image dataset. Next, we used a generalized estimating equation model with an exchangeable correlation structure at the patient level to test for differences in the specificity between the conventional and optimal image datasets. Finally, we performed 1000 iterations of the above steps and calculated the minimum sample size of patients without PDAC required to achieve 90% empirical power, which was 43 patients. The “rmvbin” function from the R package “bindata” was used to generate random numbers. Patients with PDAC were enrolled until the number of patients without PDAC reached 43, which was 67 patients. As measures of diagnostic performance, the sensitivity, specificity, positive predictive value (PPV), negative predictive value (NPV), and accuracy were calculated for each method by all radiologists. Generalized estimating equation models were used to calculate each measure by all radiologists, experts, and trainees and to compare the conventional and optimal image datasets. If the diagnosis was exactly the same on the conventional and optimal image datasets or if either index was 100%, the results were not testable; therefore, the corresponding result was marked as “Not Calculated” (N.C.). The confidence ratings were considered ordinal categorical data, and an ordinal logistic regression model was used to compare the conventional and optimal image datasets for patients with and without PDAC, respectively. To accommodate data showing within-class correlations, the variance estimated from the ordinal logistic regression model was corrected using the Huber–White sandwich estimator.

Cohen’s kappa coefficient was used to assess the inter-rater reliability of each expert and trainee diagnosis, and Fleiss’ kappa coefficient was used to assess the inter-rater reliability among all radiologists. The inter-rater reliability between expert and trainee groups was compared using the z-test under the assumption that the kappa coefficient followed an asymptotic normal distribution.

Two-sided *P* values ≤ 0.05 were considered indicative of statistical significance. The statistical analyses were performed using the R software version 4.3.1 (www.r-project.org).

## Results

### Patients’ demographics and tumor characteristics

**Fig. 1:**
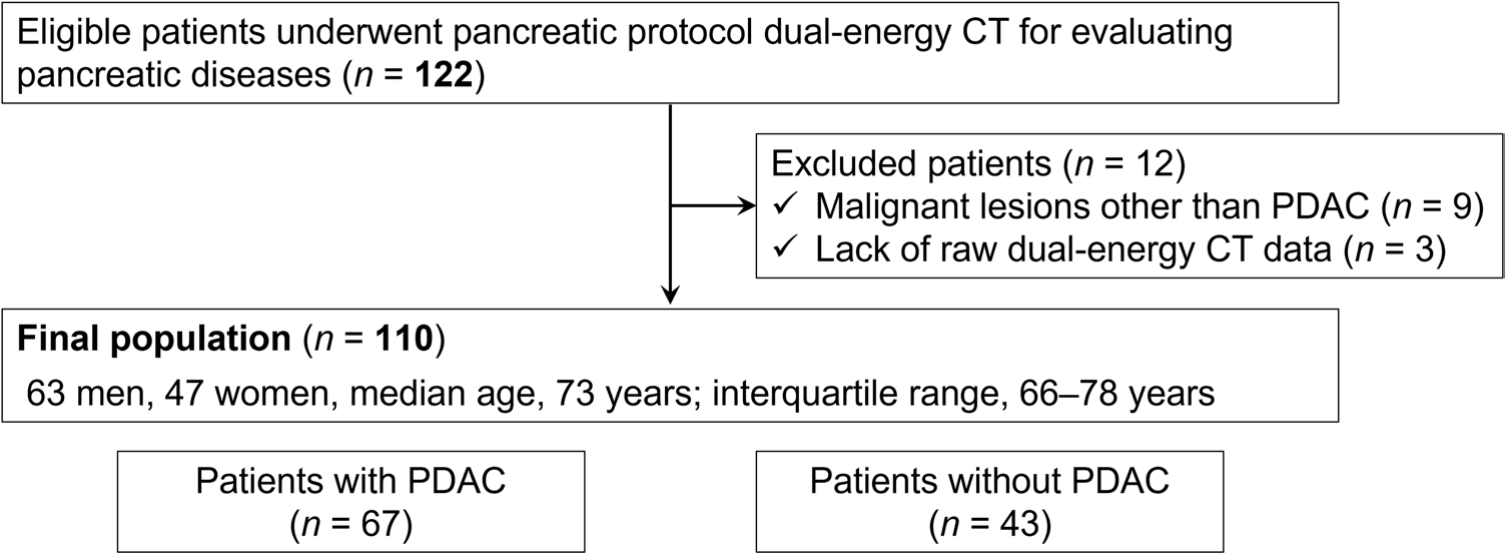
Flowchart showing the number of included and excluded patients

Among the 122 patients, 12 were excluded due to malignancy other than PDAC (*n* = 9) and lack of raw DECT data (*n* = 3). No patients who had severe pancreatic atrophy or received reduced contrast material dose were observed. The final sample consisted of 110 patients (63 men and 47 women; median age, 73 years [interquartile range, 66–78 years]). Among the 110 patients, 67 patients (61%) had pathologically proven PDAC (Fig. 1). We found no difference in the patients’ age (*P* = 0.73), sex (*P* > 0.99), height (*P* = 0.82), body weight (*P* = 0.56), and body mass index (*P* = 0.64) between patients with and without PDAC (Table 2). Clinical diagnoses and tumor characteristics are summarized in Table 2.

**Table 2:** Patients’ demographics and tumor characteristics

Characteristics	All cases	With PDAC	Without PDAC	*P* value With vs. without PDAC
Patients’ demographics				
Number of patients	110	67	43	N.A
Age (years)	73 (66–78)	73 (66–79)	71 (66–78)	0.73
Men:women	63:47	38:29	25:18	> 0.99
Height (cm)	160 (152–166)	160 (152–167)	160 (153–165)	0.82
Body weight (kg)	56 (49–65)	55 (48–64)	59 (49–67)	0.56
Body mass index (kg/m^2^)	22 (20–25)	22 (20–25)	23 (20–25)	0.64
Clinical diagnosis				N.A
Pancreatic ductal adenocarcinoma	67/110 (60)	67/67 (100)	–	
Intraductal papillary mucinous neoplasm	24/110 (22)	–	24/43 (56)	
Normal study	8/110 (7)	–	8/43 (19)	
Autoimmune pancreatitis	4/110 (4)	–	4/43 (9)	
Chronic pancreatitis	2/110 (2)	–	2/43 (5)	
Walled-off necrosis after acute pancreatitis	2/110 (2)	–	2/43 (5)	
Intrapancreatic accessory spleen	1/110 (1)	–	1/43 (2)	
Gastric gastrointestinal stromal tumor	1/110 (1)	–	1/43 (2)	
Gallbladder cancer	1/110 (1)	–	1/43 (2)	
Tumor characteristics				N.A
Tumor size (mm)	–	28 (19–37)	–	
Location (H/B/T)	–	35/14/18	–	
cT (0/1a/1b/1c/2/3/4)	–	0/0/1/18/33/9/6	–	
cN (0/1/2)	–	48/14/5	–	
cM (0/1)	–	44/23	–	
Stage (IA/IB/IIA/IIB/III/IV)	–	18/15/0/7/4/23	–	

*Note* Data are medians, with interquartile ranges in parentheses. Data are numerator/denominator, with percentages in parentheses in clinical diagnosis.

PDAC = pancreatic ductal adenocarcinoma, N.A. = not applicable, H = head, B = body, T = tail, cT = clinical T, cN = clinical N, cM = clinical M

### Identification of the optimal material decomposition images

**Fig. 2:**
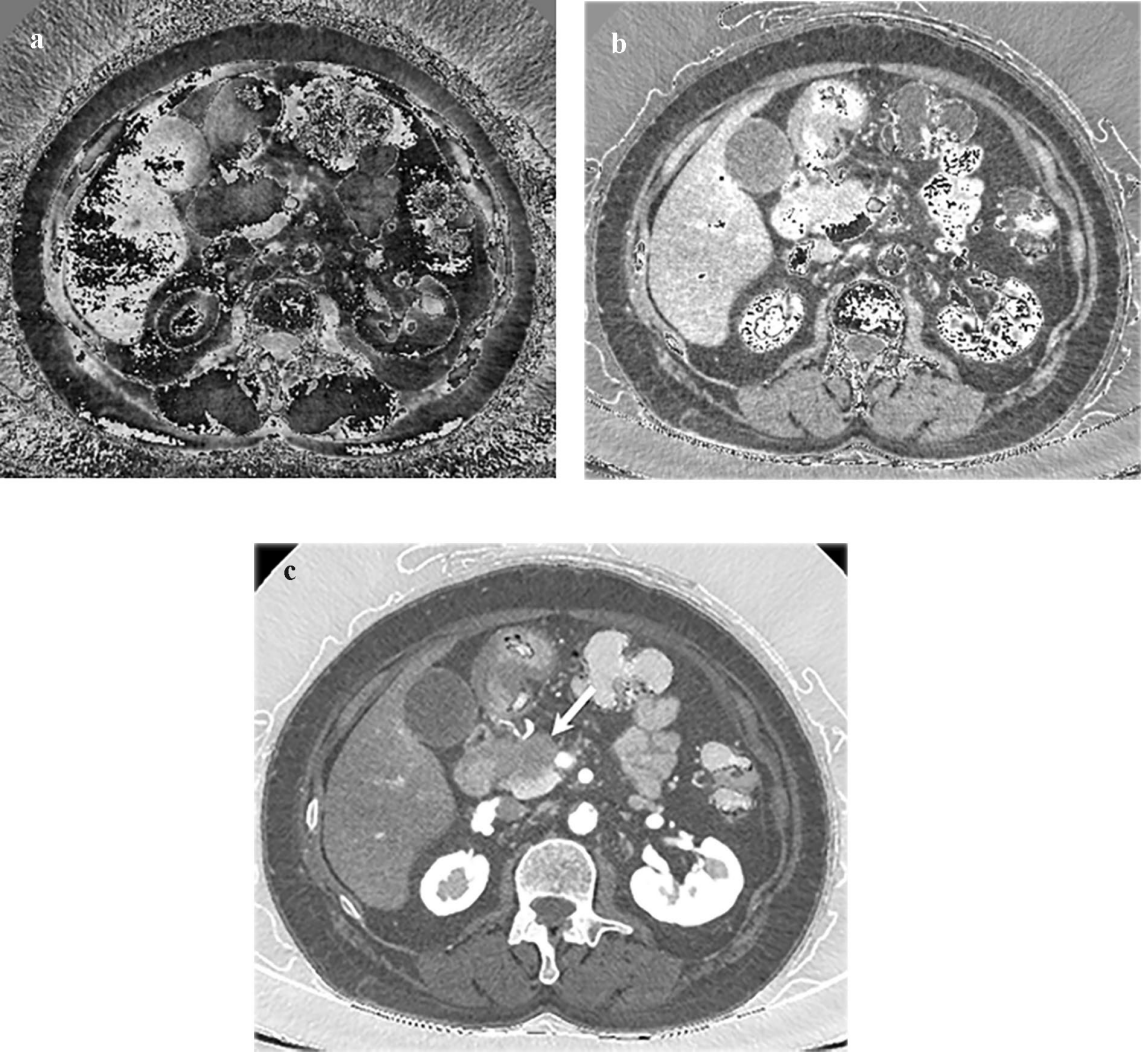
The top three image datasets showing maximum contrast difference between the normal pancreas and pancreatic ductal adenocarcinoma (PDAC). **a** Calc bone/Calc measured and **b** Monosodium urate/Blood images show marked image noise and unacceptable image quality. **c** Silicon/Struvite image shows relatively good image quality and excellent contrast difference between the normal pancreas and PDAC (arrow)

The top three MD image datasets showing maximum contrast difference between the normal pancreas and PDAC were Calc Bone/Calc Measured images (mean, 412; SD, 2295; and *z* value, 0.18), Monosodium urate/Blood images (mean, 331; SD, 1564; and *z* value, 0.21), and Silicon/Struvite images (mean, – 256; SD, 563; and *z* value, –0.46). The highest absolute figure of z value among these three image datasets was found in the Silicon/Struvite images. Additionally, the study coordinator and another three radiologists (M.A., Y.T.*,* and H.K., with 2, 6, and 25 years of experience in radiology) selected the best one among the three image datasets based on the image quality for diagnostic acceptability, including normal anatomy as well as PDAC, in consensus. The Calc Bone/Calc Measured and Monosodium urate/Blood images had marked image noise and the radiologists judged unacceptable image quality; in contrast, the Silicon/Struvite images showed relatively good image quality (Fig. 2). Therefore, we identified Silicon/Struvite images as the optimal MD images and the optimal image dataset consisted of the Silicon/Struvite images and 50-keV VMIs.

### Diagnostic performance

The sensitivity, specificity, PPV, NPV, and accuracy for diagnosing PDAC in the conventional and optimal image datasets are summarized in Table 3. Among all radiologists, the sensitivity, specificity, PPV, NPV, and accuracy in the conventional image dataset were 99%, 81%, 89%, 97%, and 92% and those in the optimal image dataset were 98%, 88%, 93%, 97%, and 94%, respectively. The specificity (*P* = 0.006), PPV (*P* < 0.001), and accuracy (*P* = 0.01) were higher in the optimal image dataset than in the conventional image dataset. We found no difference in the sensitivity (*P* = 0.34) and NPV (*P* = 0.33) between the conventional and optimal image datasets. Among the experts, the sensitivity, specificity, PPV, NPV, and accuracy were 99%, 90%, 94%, 98%, and 95% in both the conventional and optimal image datasets (*P* values cannot be calculated). Among the trainees, the sensitivity, specificity, PPV, NPV, and accuracy in the conventional image dataset were 99%, 72%, 85%, 97%, and 88% and those in the optimal image dataset were 98%, 87%, 92%, 96%, and 94%, respectively. The specificity (*P* = 0.002), PPV (*P* < 0.001), and accuracy (*P* = 0.006) were higher in the optimal image dataset than in the conventional image dataset. We found no difference in the sensitivity (*P* = 0.32) and NPV (*P* = 0.49) between the conventional and optimal image datasets. The diagnostic performance for each radiologist is shown in Table 3.

**Table 3:** Diagnostic performance for detecting pancreatic ductal adenocarcinoma in the conventional and optimal image datasets

Radiologists	Image sets	Sensitivity	Specificity	PPV	NPV	Accuracy
All radiologists	Conventional	99 (264/268)	81 (139/172)	89 (264/297)	97 (139/143)	92 (403/440)
	Optimal	98 (263/268)	88 (152/172)	93 (263/283)	97 (152/157)	94 (415/440)
	*P* value	0.34	0.006	< 0.001	0.33	0.01
Expert	Conventional	99 (132/134)	90 (77/86)	94 (132/141)	98 (77/79)	95 (209/220)
	Optimal	99 (132/134)	90 (77/86)	94 (132/141)	98 (77/79)	95 (209/220)
	*P* value	N.C	N.C	N.C	N.C	N.C
Radiologist 1	Conventional	99 (66/67)	93 (40/43)	96 (66/69)	98 (40/41)	96 (106/110)
	Optimal	99 (66/67)	93 (40/43)	96 (66/69)	98 (40/41)	96 (106/110)
	*P* value	N.C	N.C	N.C	N.C	N.C
Radiologist 2	Conventional	99 (66/67)	86 (37/43)	92 (66/72)	97 (37/38)	94 (103/110)
	Optimal	99 (66/67)	86 (37/43)	92 (66/72)	97 (37/38)	94 (103/110)
	*P* value	N.C	N.C	N.C	N.C	N.C
Trainee	Conventional	99 (132/134)	72 (62/86)	85 (132/156)	97 (62/64)	88 (194/220)
	Optimal	98 (131/134)	87 (75/86)	92 (131/142)	96 (75/78)	94 (206/220)
	*P* value	0.32	0.002	< 0.001	0.49	0.006
Radiologist 3	Conventional	97 (65/67)	79 (34/43)	88 (65/74)	94 (34/36)	90 (99/110)
	Optimal	96 (64/67)	88 (38/43)	93 (64/69)	93 (38/41)	93 (102/110)
	*P* value	0.32	0.04	0.02	0.42	0.18
Radiologist 4	Conventional	100 (67/67)	65 (28/43)	82 (67/82)	100 (28/28)	86 (95/110)
	Optimal	100 (67/67)	86 (37/43)	92 (67/73)	100 (37/37)	95 (104/110)
	*P* value	N.C	0.001	< 0.001	N.C	0.003

*Note* Data are percentages, with numerator/denominator in parentheses

PPV = positive predictive value, NPV = negative predictive value, N.C. = not calculated

### Confidence ratings for diagnosing PDAC

**Fig. 3:**
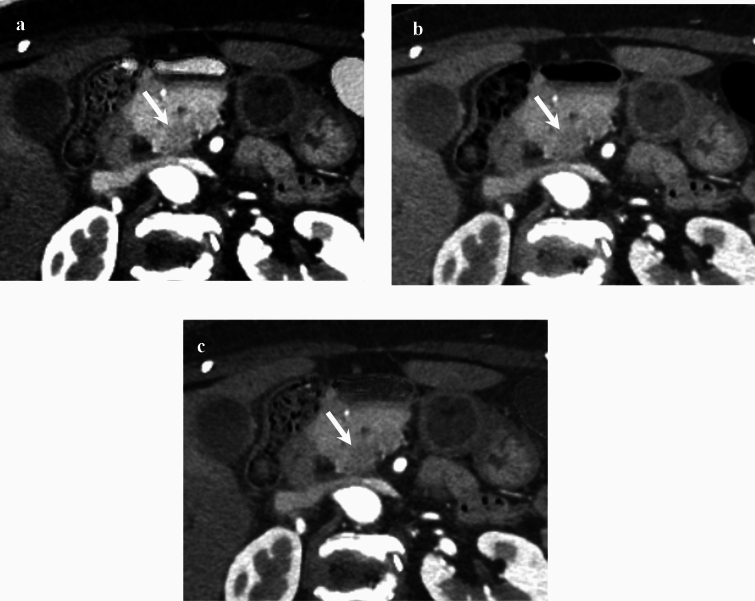
An 80-year-old woman with pancreatic ductal adenocarcinoma (PDAC) at the pancreatic head (arrow). Axial **a** Silicon/Struvite image (contrast difference, – 164%) shows better PDAC conspicuity compared to **b** 50-keV virtual monoenergetic (contrast difference, 27%) and **c** iodine-based images (contrast difference, 24%)

The confidence ratings for diagnosing PDAC are summarized in Table 4. The improved diagnostic confidence was determined when the confidence rating was higher in the optimal image dataset than in the conventional image dataset in patients with PDAC and lower in patients without PDAC. The diagnostic confidences in patients with PDAC among all, experts, and trainees (*P* < 0.001 for all) (Fig. 3), and those without PDAC among all (*P* < 0.001), experts (*P* = 0.03), and trainees (*P* = 0.002) were improved in the optimal image dataset compared with the conventional image dataset. The confidence ratings for each radiologist are shown in Table 4.

**Table 4:** Confidence ratings for diagnosing pancreatic ductal adenocarcinoma

Radiologists	Confidence rating	With PDAC			Without PDAC		
		Conventional	Optimal	*P* value	Conventional	Optimal	*P* value
All radiologists	1	0/268 (0)	1/268 (0)	< 0.001	34/172 (19)	42/172 (24)	< 0.001
	2	4/268 (2)	4/268 (2)		105/172 (61)	110/172 (64)	
	3	55/268 (20)	20/268 (7)		29/172 (17)	13/172 (8)	
	4	104/268 (39)	115/268 (43)		3/172 (2)	6/172 (3)	
	5	105/268 (39)	128/268 (48)		1/172 (1)	1/172 (1)	
Expert	1	0/134 (0)	1/134 (1)	< 0.001	27/86 (32)	32/86 (37)	0.03
	2	2/134 (2)	1/134 (1)		50/86 (58)	45/86 (53)	
	3	33/134 (24)	12/134 (9)		7/86 (8)	6/86 (7)	
	4	43/134 (32)	51/134 (38)		1/86 (1)	2/86 (2)	
	5	56/134 (42)	69/134 (51)		1/86 (1)	1/86 (1)	
Radiologist 1	1	0/67 (0)	0/67 (0)	< 0.001	14/43 (33)	17/43 (40)	0.09
	2	1/67 (2)	1/67 (2)		26/43 (60)	23/43 (54)	
	3	21/67 (31)	6/67 (9)		2/43 (5)	1/43 (2)	
	4	19/67 (28)	29/67 (43)		0/43 (0)	1/43 (2)	
	5	26/67 (39)	31/67 (46)		1/43 (2)	1/43 (2)	
Radiologist 2	1	0/67 (0)	1/67 (2)	< 0.001	13/43 (30)	15/43 (35)	0.16
	2	1/67 (2)	0/67 (0)		24/43 (56)	22/43 (51)	
	3	12/67 (18)	6/67 (9)		5/43 (12)	5/43 (12)	
	4	24/67 (36)	22/67 (33)		1/43 (2)	1/43 (2)	
	5	30/67 (44)	38/67 (56)		0/43 (0)	0/43 (0)	
Trainee	1	0/134 (0)	0/134 (0)	< 0.001	7/86 (8)	10/86 (12)	0.002
	2	2/134 (2)	3/134 (2)		55/86 (64)	65/86 (75)	
	3	22/134 (16)	8/134 (6)		22/86 (26)	7/86 (8)	
	4	61/134 (45)	64/134 (48)		2/86 (2)	4/86 (5)	
	5	49/134 (37)	59/134 (44)		0/86 (0)	0/86 (0)	
Radiologist 3	1	0/67 (0)	0/67 (0)	0.005	7/43 (16)	10/43 (23)	0.02
	2	2/67 (3)	3/67 (5)		27/43 (63)	28/43 (65)	
	3	6/67 (9)	2/67 (3)		8/43 (19)	2/43 (5)	
	4	39/67 (58)	37/67 (55)		1/43 (2)	3/43 (7)	
	5	20/67 (30)	25/67 (37)		0/43 (0)	0/43 (0)	
Radiologist 4	1	0/67 (0)	0/67 (0)	< 0.001	0/43 (0)	0/43 (0)	0.002
	2	0/67 (0)	0/67 (0)		28/43 (65)	37/43 (86)	
	3	16/67 (24)	7/67 (10)		14/43 (33)	5/43 (12)	
	4	22/67 (33)	34/67 (51)		1/43 (2)	1/43 (2)	
	5	29/67 (43)	26/67 (39)		0/43 (0)	0/43 (0)	

*Note* Data are numerator/denominator, with percentages in parentheses

PDAC = pancreatic ductal adenocarcinoma

### Interobserver variability

All radiologists showed moderate agreement in the conventional (*ĸ*, 0.43; 95% confidence interval [CI]: 0.39, 0.47) and optimal (*ĸ*, 0.47; 95% CI: 0.43, 0.52) image datasets. We found no difference in kappa values between experts and trainees for the conventional (*ĸ*, 0.52 vs. 0.45; *P* = 0.16) and optimal (*ĸ*, 0.52 vs. 0.52; *P* = 0.48) image datasets.

## Discussion

Iodine-based MD images are becoming increasingly used in the diagnosis of pancreatic lesions, in particular PDAC [5]. Although iodine-based MD images has been reported to be able to improve tumor conspicuity [7], its impact on diagnostic performance has not been evaluated. Additionally, a DECT acquisition enables the generation of numerous MD image datasets which could potentially improve diagnosis compared to iodine-based images; however, this has not been considered either. In our study, we initially identified Silicon/Struvite images as the optimal MD images for diagnosing PDAC. When used in combination with the conventional 50-keV VMIs, the Silicon/Struvite images performed better than iodine-based images with improved diagnostic performance for detecting PDAC especially for trainees. Although the diagnostic performance was comparable among experts, diagnostic confidence for detecting PDAC was improved with the new MD image datasets compared to the conventional image dataset.

VMIs at lower energy levels have shown to have improved diagnostic performance for detecting PDAC. Noda et al. [4] reported that the accuracy was improved on 40-keV VMIs from 81% to 85% compared with 70-keV VMIs. We adopted 50-keV VMIs, while not 40-keV, as the conventional image dataset for the current study as it was consistent with our clinical practice. The conventional image dataset did demonstrate an accuracy of 92% as was higher than previous study which assessed only using 40-keV VMIs [4]. Although no study has yet proven the clinical impact of only iodine-based images on diagnostic performance in PDAC, a previous study demonstrated that iodine-based images and VMIs at around 50-keV increase PDAC conspicuity [9], and we believed that this led high-diagnostic performance as demonstrated in our study. Therefore, even though the conventional image dataset used in daily clinical practice is already a fairly accurate diagnostic tool, we believe that the optimal image dataset is likely to be useful, because it surpasses it.

In our study, we identified Silicon/Struvite images from 702 MD image datasets which surpass iodine-based images in diagnostic performance for detecting PDAC. The limitation of two material basis is that since the tissues, normal, or tumor has complex composition, it is difficult to estimate optimal basic material pair in advance. Inversely, material density was calculated comprehensively for all available material pairs in this study and the material pair which maximize the difference between the pancreas and PDAC was selected retrospectively. Silicon/Struvite was selected as the result of this calculation model, although it was understandable that silicon and struvite are not major composition material in human body. In two MD, the linear attenuation coefficient derived from the CT attenuation is expressed as a linear combination of the mass attenuation coefficients and densities of two basis materials (e.g., iodine and water). This process involves solving a system of two linear equations, using attenuation coefficients at high and low energies to estimate the densities of the two materials. The operation can be interpreted as a coordinate transformation from the space defined by attenuation coefficients at two energies to the space defined by the mass attenuation coefficients of the basis materials. Maximizing the contrast difference is equivalent to maximizing the difference in material density between ROIs, such as pancreas and PDAC. This can also be interpreted as the slope of a line connecting two ROIs in the attenuation coefficient space being similar to the direction of one of the coordinate axes in the mass attenuation coefficient space. In this study, Silicon/Struvite images were selected as the best material pair. It is considered that the direction of the silicon mass attenuation coefficient axis is close to that of the line connecting the pancreas and PDAC.

A combination of Silicon/Struvite images along with the 50-keV VMIs demonstrated higher specificity, PPV, and accuracy for diagnosing PDAC while maintaining the sensitivity and NPV compared to the iodine-based images and 50-keV VMIs. The improved performance of Silicon/Struvite images are likely due to higher contrast compared to iodine-based images. Interestingly, the use of Silicon/Struvite images enhanced the performance of trainees to the level of experts (94% vs. 95%). In addition, the confidence for denying the existence of PDAC was improved with the Silicon/Struvite image datasets. Pancreas is often prone to parenchymal contrast irregularities due to inflammation, etc. Thus, we believe that the trainees were pointing out trivial parenchymal contrast irregularities as positive findings, but Silicon/Struvite images have made it possible to dismiss them thanks to the increased contrast of the positive findings. In the experts, the diagnostic performance was completely comparable between the conventional and optimal image datasets. While the diagnostic performance remained unchanged, the confidence in diagnosis of PDAC was significantly improved with the Silicon/Struvite image datasets. Especially when focusing on patients with PDAC, equivocal cases were reduced from 24% to 9% in experts by reviewing the optimal image dataset.

Our study had several limitations. First, rapid kilovoltage-switching DECT scanner from a single vendor and only 27 materials were used, which may have introduced an element of bias. Second, the processes for identification of the optimal MD images for diagnosing PDAC and the window settings for the iodine-based and Silicon/Struvite images have not been optimized. Finally, we used a contrast material of 600-mg of iodine per kg and adaptive statistical iterative reconstruction-Veo of 40% in this study. The iodine dosage and image reconstruction method could affect the image contrast and noise. However, the optimal iodine dosage and reconstruction method are still controversial in DECT scanning.

In conclusion, we identified the Silicon/Struvite images as the optimal material decomposition (MD) images for diagnosing pancreatic ductal adenocarcinoma (PDAC) from 702 MD image datasets. The Silicon/Struvite images in combination with 50-keV virtual monoenergetic images (VMIs) enabled a more accurate diagnosis of PDAC, along with higher reader confidence compared to the combination of iodine-based images and 50-keV VMIs.
